# ROBOTIC-ASSISTED VERSUS LAPAROSCOPIC INCISIONAL HERNIA REPAIR: DIFFERENCES IN DIRECT COSTS FROM A BRAZILIAN PUBLIC INSTITUTE PERSPECTIVE

**DOI:** 10.1590/0102-672020220002e1714

**Published:** 2023-01-09

**Authors:** Thiago Nogueira COSTA, Francisco TUSTUMI, Lucas Sousa Maia FERROS, Bárbara Buccelli COLONNO, Ricardo Zugaib ABDALLA, Ulysses RIBEIRO-JUNIOR, Ivan CECCONELLO

**Affiliations:** 1 Universidade de São Paulo, Department of Gastroenterology – São Paulo (SP), Brazil.

**Keywords:** Incisional Hernia, Minimally Invasive Surgical Procedures, Herniorrhaphy, Robotic Surgical Procedures, Hérnia Incisional, Procedimentos Cirúrgicos Minimamente Invasivos, Herniorrafia, Procedimentos Cirúrgicos Robóticos

## Abstract

**BACKGROUND:**

Robotic-assisted surgery research has grown dramatically in the past two decades and the advantages over traditional videolaparoscopy have been extensively debated. For hernias, the robotic system can increase intraoperative strategies, especially in complex hernias or incisional hernias.

**AIMS:**

This study aimed to compare the direct cost differences between robotic and laparoscopic hernia repair and determine each source of expenditure that may be related to the increased costs in a robotic program from the perspective of a Brazilian public institution.

**METHODS:**

This study investigated the differences in direct costs from the data generated from a trial protocol (ReBEC: RBR-5s6mnrf). Patients with incisional hernia were randomly assigned to receive laparoscopic ventral incisional hernia repair (LVIHR) or robotic ventral incisional hernia repair (RVIHR). The direct medical costs of hernia treatment were described in the Brazilian currency (R$).

**RESULTS:**

A total of 19 patients submitted to LVIHR were compared with 18 submitted to RVIHR. The amount spent on operation room time (RVIHR: 2,447.91±644.79; LVIHR: 1,989.67±763.00; p=0.030), inhaled medical gases in operating room (RVIHR: 270.57±211.51; LVIHR: 84.55±252.34; p=0.023), human resources in operating room (RVIHR: 3,164.43±894.97; LVIHR: 2,120.16±663.78; p<0.001), material resources (RVIHR: 3,204.32±351.55; LVIHR: 736.51±972.32; p<0.001), and medications (RVIHR: 823.40±175.47; LVIHR: 288.50±352.55; p<0.001) for RVIHR was higher than that for LVIHR, implying a higher total cost to RVIHR (RVIHR: 14,712.24±3,520.82; LVIHR: 10,295.95±3,453.59; p<0.001). No significant difference was noted in costs related to the hospital stay, human resources in intensive care unit and ward, diagnostic tests, and meshes.

**CONCLUSION:**

Robotic system adds a significant overall cost to traditional laparoscopic hernia repair. The cost of the medical and robotic devices and longer operative times are the main factors driving the difference in costs.

## RESUMO

**RACIONAL:**

A pesquisa em cirurgia robótica assistida cresceu dramaticamente nas últimas duas décadas e as vantagens sobre a videolaparoscopia tradicional têm sido amplamente debatidas. Para as hérnias, o sistema robótico pode aumentar as estratégias intraoperatórias, principalmente em hérnias complexas ou hérnias incisionais.

**OBJETIVOS:**

Comparar as diferenças de custo direto entre a hernioplastia incisional robótica e a laparoscópica e determinar cada fonte de gasto que pode estar relacionada ao aumento de custos em um programa de robótica na perspectiva de uma instituição pública brasileira.

**MÉTODOS:**

Investigar as diferenças nos custos diretos dos dados gerados a partir de um protocolo de ensaio clínico (ReBEC: RBR-5s6mnrf). Pacientes com hérnia incisional foram aleatoriamente designados para serem submetidos a reparo robótico ou laparoscópico (RVIHR). Os custos foram descritos na moeda brasileira (R$).

**RESULTADOS:**

Dezenove pacientes submetidos à cirurgia robótica foram comparados com dezoito submetidos à cirurgia laparoscópica. O valor gasto com tempo de centro cirúrgico (Robótica: 2.447,91±644,79; Robótica: 1.989,67±763,00; p=0,030), gases medicinais inalados em centro cirúrgico (Robótica: 270,57±211,51; Robótica: 84,55±252,34; p=0,023), recursos humanos em centro cirúrgico (Robótica: 3.164,43±894,97; Laparoscópica: 2.120,16±663,78; p<0,001), recursos materiais (Robótica : 3.204,32±351,55; Robótica: 736,51±972,32; p<0,001) e medicamentos (Robótica: 823,40±175,47; Robótica: 288,50 ± 352,55; p<0,001) para cirurgia robótica foi maior que cirurgia laparoscópica, implicando em maior custo total para cirurgia robótica (Robótica: 14.712,24±3.520,82; Laparoscópica: 10.295,95±3.453,59; p<0,001). Não foi observada diferença significativa nos custos relacionados à permanência hospitalar, recursos humanos em UTI e enfermaria, exames diagnósticos e telas.

**CONCLUSÕES:**

O sistema robótico adiciona um custo global significativo à hernioplastia incisional laparoscópica tradicional. O custo dos dispositivos médicos e robóticos, além de tempos cirúrgicos mais prolongados, são os principais fatores que impulsionam a diferença nos custos.

## INTRODUCTION

Robotic-assisted surgery research has grown dramatically in the past two decades, leveraged by top Gross Domestic Product (GDP) countries^9^. A bibliometric analysis demonstrated the growth of 573% of robotic surgery articles published in the past decade^9^. The advantages of robotic platform surgeries over traditional videolaparoscopy have been extensively debated^11,13,14,16,24,25,28^. For hernias, the robotic system can increase intraoperative strategies, especially in complex hernias or incisional hernias^2,8^. The benefits comprise high-quality 3D visualization of the abdominal cavity, gain in movement allowing easier dissection of multiple adhesions, the release of the rectus muscle, intraperitoneal mesh suturing, and complex reconstruction of the abdominal wall^7^.

Several comparative studies of robotic-assisted primary, inguinal, or incisional ventral hernia repair versus laparoscopy found no significant differences in postoperative outcomes^5,6,10,15,21-23^. Both robotic and laparoscopic hernia repair approaches are equally effective for postoperative recovery compared with open surgery^15^. In the first randomized controlled trial comparing robotic versus laparoscopic incisional ventral hernia repair in Brazil, we also found no evidence of differences in hospitalization, surgical complications, and recurrence rate. However, robotic surgery has nearly doubled operating room (OR) time^10^.

The first robotic systems acquired in Brazil date back to 2007, covering private institutions. In 2012, public hospitals in Brazil acquired this system, making surgical technology available to patients treated in the public health system^18,20^. The Brazilian Public Health System (SUS) is the result of the Health Reform Movement, which culminated in the creation of the Unified Health System (in Portuguese: *Sistema Único de Saúde* — SUS) based on the principles of universality, integrality, and equality. Despite its implementation, its process is considered unfinished, and with deviations^3^. The relationship between funding and the system’s care model is one of the biggest obstacles to the deployment of expensive new treatments, such as the robotic system. In this aspect, it is essential to know better the cost impacts of a public health system robotic program. Consequently, this study aimed to compare the direct cost differences between robotic and laparoscopic hernia repair surgery and determine each source of expenditure that may be related to the increased costs in a robotic program from the perspective of a Brazilian public institution.

## METHODS

### Study design and participants

This study investigated the differences in direct costs from the data generated from a randomized trial protocol (Brazilian Registry of Clinical Trials, ReBEC; ID: RBR-5s6mnrf). The clinical outcomes of this protocol were previously reported^5^.

All hernia repairs took place at *Instituto do Câncer do Estado de São Paulo* (ICESP), where the robot-assisted program was implemented in 2015. ICESP is a public Brazilian cancer institute located in Sao Paulo and is supported by the National Healthcare System (SUS). Recruitment was performed in 2015, and patients were followed up for 2 years. All patients were treated for incisional hernia following open oncologic surgery.

### Ethical aspects

The local Ethics Committee approved the study protocol (CAAE: 40789014.3.0000.0065), and all patients signed a written informed consent form.

### Costs estimation

The direct medical costs of hernia treatment are described from the Institute’s perspective. A mixed methodology of micro-costing and apportionment of the macro-costing was used. The estimate for daily costs related to hospitalization and surgery (OR time, medical and multidisciplinary consultations, daily charges in hospital wards, intensive care units [ICUs], and ambulatory visits) were valued by apportioning fixed and variable costs (of human resources, material resources, and infrastructure) to assess the respective unit values of the Institute health care service costs used. Drugs, medical devices, nutrition, blood, laboratory, and imaging studies were valued by micro-costing calculation according to individual patient consumption multiplied by the respective acquisition cost. The OR time was measured from entry until the patient’s departure from the OR, including the anesthesia and surgery. The mean cost of laparoscopic ventral incisional hernia repair (LVIHR) and robotic ventral incisional hernia repair (RVIHR) was calculated by averaging patient costs for each group. Costs were expressed in the Brazilian currency (Real, R$). In May 2015, R$1.00=US$ 0.33.

### Interventional and control groups

Patients with an incisional hernia were randomly assigned to receive any interventions: LVIHR or RVIHR, with either an intraperitoneal onlay mesh (IPOM) or a Rives-Stoppa procedure. All robotic-assisted procedures performed in this study used the da Vinci Si platforms. When possible, any defect was closed using a unidirectional suture. An intraperitoneal-coated macroporous multifilament polyester mesh was placed at least 5 cm overlap in all directions to cover the original hernia size.

### Statistical analysis

Qualitative variables were described as counts and percentages. Continuous variables were described as mean, median, standard deviation, and 95% confidence intervals (95%CIs). Differences between groups were assessed by the Mann-Whitney U test for continuous variables and Fisher’s exact test or chi-square test for categorical variables. Statistical analyses were performed with the STATA software, version 16.0 (StataCorp LLC).

## RESULTS

In the study, 19 patients submitted to LVIHR were compared with 18 patients submitted to RVIHR.

**Figure 1:**
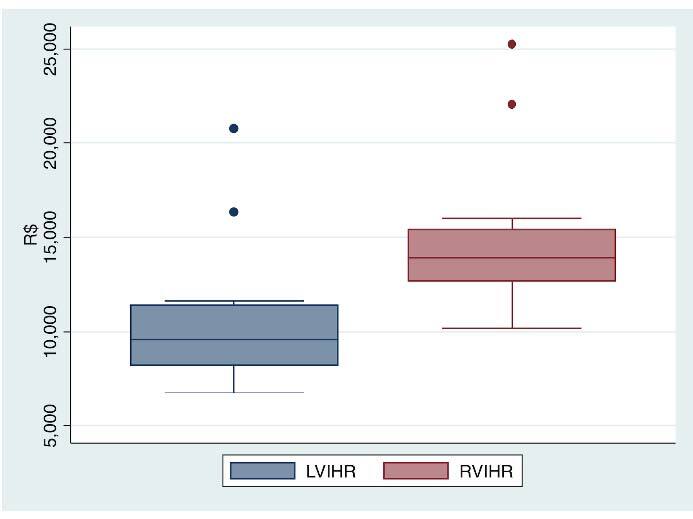
Total costs per patient (mean and standard deviation) between robotic ventral incisional hernia repair and laparoscopic ventral incisional hernia repair.

No significant difference was noted in costs related to hospital stay (RVIHR: 1,641.50±767.85; LVIHR: 1,749.61±1,130.48; p=0.738), inhaled medical gases (ICU) (RVIHR: 0.00; LVIHR: 10.83±44.92; p=0.331), inhaled medical gases (ward) (RVIHR: 0.00; LVIHR: 10.79±44.75; p=0.331), human resources (ICU) (RVIHR: 0.00; LVIHR: 104.60±433.68; p=0.331), human resources (ward) (RVIHR: 1,320.43±632.22; LVIHR: 1,443.11±948.26; p=0.648), diagnostic tests (RVIHR: 48.69±80.10; LVIHR 82.81±157.66; p 0.421), and prosthesis, meshes, and special devices (RVIHR: 1,790.98±2,023.45; LVIHR: 1,674.82±594.17; p=0.810). Meantime, the amount spent on operation room time (RVIHR: 2,447.91±644.79; LVIHR: 1,989.67±763.00; p=0.030), inhaled medical gases (OR) (RVIHR: 270.57±211.51; LVIHR: 84.55±252.34; p*=*0.023), human resources (OR) (RVIHR: 3,164.43±894.97; LVIHR: 2,120.16±663.78; p<0.001), material resources (RVIHR: 3,204.32±351.55; LVIHR: 736.51±972.32; p<0.001), medications (RVIHR: 823.40±175.47; LVIHR: 288.50±352.55; p<0.001) for RVIHR was significantly higher than that for LVIHR, implying a much higher total cost to the patient in RVIHR than in LVIHR (RVIHR: 14,712.24±3520.82; LVIHR: 10,295.95±3,453.59; p<0.001) (Figure 1, Table 1).

**Table 1:** Mean costs differences between robotic ventral incisional hernia repair and laparoscopic ventral incisional hernia repair.

Costs (R$)	RVIHR		LVIHR		Mean difference	p-value
	Mean	SD	Mean	SD		
Hospital stay	1,641.50	767.85	1,749.61	1,130.48	-108.11	0.738
OR time	2,447.91	644.79	1,989.67	763.00	458.24	0.030
Inhaled medical gases (OR)	270.57	211.51	84.55	252.34	186.02	0.023
Inhaled medical gases (ICU)	0.00	0.00	10.83	44.92	-10.83	0.331
Inhaled medical gases (ward)	0.00	0.00	10.79	44.75	-10.79	0.331
Human resources (OR)	3,164.43	894.97	2,120.16	663.78	1,044.28	<0.001
Human resources (ICU)	0.00	0.00	104.60	433.68	-104.60	0.331
Human resources (ward)	1,320.43	632.22	1,443.11	948.26	-122.67	0.648
Diagnostic tests	48.69	80.10	82.81	157.66	-34.12	0.421
Material resources*	3,204.32	351.55	736.51	972.32	2467.82	<0.001
Prosthesis, meshes, and special devices**	1,790.98	2,023.45	1,674.82	594.17	116.15	0.810
Medications	823.40	175.47	288.50	352.55	534.90	<0.001
Total costs per patient	14,712.24	3,520.82	10,295.95	3,453.59	4,416.28	<0.001

Costs were expressed as Real (R$), the monetary unit of Brazil. RVIHR=robotic ventral incisional hernia repair; LVIHR=laparoscopic ventral incisional hernia repair; OR: operation room; ICU: intensive care unit; SD: standard deviation.

To investigate the medications costs difference between RVIHR and LVIHR, we analyzed the drug vials consumed per patient (Table 2).

**Table 2:** Drug vials consumed per patient.

	RVIHR		LVIHR		p-value
	Mean	SD	Mean	SD	
Routine medications	8.6	7.62	8.53	6.92	0.888
Intravenous fluids	17.8	11.01	24.11	17.79	0.089
Electrolyte replacement	1.8	1.94	2.37	1.8	0.191
Anesthetics	5.55	1.54	4.26	1.88	0.016
Local anesthetic agents	4.5	1.19	1.68	1.42	<0.001
Neuromuscular blocking agents	3.25	1.21	2.21	1.44	0.016
Neuromuscular blockade reversal agents	1.4	0.6	2.21	2.25	0.612
Vasoactive drugs	0.3	0.57	0.68	0.95	0.144
Antibiotics	2.47	7.78	4.6	1.35	0.497
Analgesics	24.7	13.5	25.58	19.61	0.966
Antiemetics	5.6	5.79	6.53	6	0.24
Other	1.5	1.19	3.53	7.28	0.885

SD=standard deviation; RVIHR=robotic ventral incisional hernia repair; LVIHR=laparoscopic ventral incisional hernia repair.

Regarding drug vials consumed per patient, no significant difference was noted in routine medications (RVIHR: 8.6±7.62 vials; LVIHR: 8.53±6.92 vials; p=0.888), intravenous fluids (RVIHR: 17.8±11.01 vials; LVIHR: 24.11±17.79 vials; p=0.089), electrolyte replacement (RVIHR: 1.8±1.94 vials; LVIHR: 2.37±1.8 vials; p=0.191), neuromuscular blockade reversal agents (RVIHR: 1.4±0.6 vials; LVIHR: 2.21±2.25 vials; p=0.612), vasoactive drugs (RVIHR: 0.3±0.57 vials; LVIHR: 0.68±0.95 vials; p=0.144), antibiotics (RVIHR: 2.47±7.78 vials; LVIHR: 4.6±1.35 vials; p=0.497), analgesics (RVIHR: 24.7±13.5 vials; LVIHR: 25.58±19.61 vials; p=0.966), antiemetics (RVIHR: 5.6±5.79 vials; LVIHR: 6.53±6 vials; p=0.24), and other (RVIHR: 1.5±1.19 vials; LVIHR: 3.53±7.28 vials; p=0.885). On the other hand, there was a significant difference in the consume of anesthetics (RVIHR: 5.55±1.54 vials; LVIHR: 4.26±1.88 vials; p=0.016), local anesthetic agents (RVIHR: 4.5±1.19 vials; LVIHR: 1.68±1.42 vials; p<0.001), and neuromuscular blocking agents (RVIHR: 3.25±1.21 vials; LVIHR: 2.21±1.44 vials; p=0.016).

## DISCUSSION

The outcome of the present study showed that comparing RVIHR and LVIHR, there is a significantly higher average cost in robotic-assisted use. These costs are mainly associated with prolonged surgical time, higher consumption of anesthetics, high mobilization of human resources, and, evidently, material devices related to the robotic machine. Knowing the costs of each variable related to the surgical intervention is essential to better allocate resources and to depict a detailed and precise budget impact analysis before implementing a robotic program in a public health institution.

Beyond material resources, the robotic system demands higher costs related to human resources. In theory, the robotic system demands only one surgeon for each surgery, decreasing the need for other surgeons during the procedures. In the United States, the physician assistant, a mid-level health care provider, may act as a bedside assistant and helps position the patient and docking, decreasing the costs related to robot-assisted procedures^19^. However, the Brazilian National Medical Board imposes that any surgery (robot-assisted or not) should be performed by at least two surgeons. Consequently, in Brazilian robotic surgeries, a physician assistant is not an alternative to reduce costs in robotics, and LVIHR and RVIHR shall have similar personal costs related to surgeons.

Due to the prolonged OR time and the complexity of the procedure, there is a demand for more number and more qualified professionals during robotic surgery. Nurses in robot-assisted surgery need to have high technical proficiency and active attitudes. Their roles include scheduling, checking for supplies, system operating, administration of circulating nurses, patient and console positioning, placing robotic arms, robotic arm sterile draping, configuring equipment and instruments, docking, and undocking^24,27^. Consequently, the complexity of the robotic OR requires comprehensive and continuous training of the robotic nurses and, frequently, more professionals than the traditional laparoscopic approach^26^. Robotic machine failure or malfunction can result in delay and prolonged operating times, and robotic nurses should correct and prompt identify the system failure, report, and take quick and suitable measures^12^. Robotic system-related material resources are expensive, and careless handling may lower the life span of robotic machine apparatus, increasing the material costs. In this setting, robotic nurses’ training can reduce the demand for an inflated number of professionals and devices in the OR, reduce operating time, and consequently lower costs^19,24,26^. All robotic teams, including surgeons, bedside assistants, nurses, and the engineer team, should be continuously trained to improve surgical outcomes and mitigate costs.

Prolonged surgical time implicates higher demand for more professionals in OR and expenditure with the OR time. Besides, prolonged surgical time implies more use of anesthetics and neuromuscular blockade agents. Anesthesia costs usually represent a minority proportion of the perioperative costs^17^. However, even being a low cost compared to the human and material resources, the drug expenses in robotic system impose a significant additional cost compared with laparoscopic hernia repair. Besides, some technical difficulties during LVIHR, such as field visualization and structures mobilization, frequently can be easily managed by adjusting bed inclination or lateralization. Nonetheless, the difficulty in changing the patient’s position after docking may compel a continuous deep neuromuscular blockage for suitable field visualization and contribute to the higher consumption of neuromuscular blockade agents in the RVIHR group.

Recent studies demonstrated the higher costs of the RVIHR compared with LVIHR. Nationwide American studies^1,4^ showed that RVIHR has higher costs than LVIHR and open hernia repair. Khoraki et al.^13^, in a retrospective study, showed that the added cost related to the robotic system was $3,106 per patient. Olavarria et al.^21^, in a multicenter controlled trial comparing RVIHR with LVIHR, the cost ratio was 1.21 (95%CI 1.07–1.38). These previously quoted studies evaluated only the global health care costs in North-American robotic centers. Abdelmoaty et al.^1^ did a more detailed cost analysis and grouped costs into fixed, personnel, medical device, and variable costs. Each of these costs was significantly higher for robot-assisted surgery than for laparoscopic. However, the authors evaluated only inguinal hernia repair. Zayan et al.^29^ included both inguinal and ventral hernia repair and grouped costs in direct and indirect. The authors showed that only direct costs were significantly higher for robotic surgery than laparoscopic, yet their methodology for cost estimation was less depicted.

All the studies quoted in the last paragraph were North American, and worldwide extrapolations are questionable. The present study gives a picture of a robotic program from a public institution in a middle-income country. Nonetheless, this study has several limitations. First, it was conducted in a single center, lacking external validity from other middle and low-income countries. In addition, only direct costs were accounted for, and costs related to the rehabilitation facility, days off work after surgery, and their impact on quality of life were not considered. Another inherent issue with any cost-analysis study is due to the fluctuation of the exchange rate over time, making it difficult to obtain a definitive analysis of the costs involved in any longitudinal study. Well-designed future cost-effectiveness and cost-utility studies can answer whether the high costs of robot-assisted approach are justifiable for all countries. Economic studies evaluating the robotic systems’ budgetary impact on health systems are crucial for determining their utility in public programs in developing or underdeveloped countries.

The present study’s findings raise the question: “Is there a role for the RVIHR in a public health system?” The answer is “yes.” If a robotic program does not root in assistance, there will be a significant delay in the dissemination of trained robotic teams and experienced surgeons and professionals worldwide, and consequently, expenses lowering tend to linger. Nayeemuddin et al.^19^ defined “surgeons and nurses” robotic training as the main modifying factors of the cost equation in robotics. Medical residency and robotics fellowship programs must be prepared and well-trained for this new world of robotic surgery and its evolution^18,19^. The benefits of robotic surgery, including visualization, increased degrees of freedom, and ergonomics^18^, must be incorporated worldwide, including in low- and middle-income countries, and the cost differences between robotic and laparoscopic procedures should be overcome. Identifying each source of expenditure that may be related to the increased costs in a robotic program from the perspective of a public institution in a middle-income country, such as Brazil, may help propose new strategies to facilitate robotic program dissemination worldwide.

## CONCLUSIONS

A robotic system adds a significant overall cost to traditional laparoscopic hernia repair. The cost of the medical and robotic devices and longer operative times are the main factors driving the difference in costs. These costs should be well known before starting any robotic public program.
